# A slotted plus-shaped antenna with a DGS for 5G Sub-6 GHz/WiMAX applications

**DOI:** 10.1016/j.heliyon.2022.e12040

**Published:** 2022-12-05

**Authors:** Liton Chandra Paul, Sajeeb Chandra Das, Tithi Rani, S.M. Muyeen, Sk. A. Shezan, Md. Fatin Ishraque

**Affiliations:** a Department of EECE, Pabna University of Science and Technology, Bangladesh; b Department of ETE, Rajshahi University of Engineering and Technology, Bangladesh; c Department of Electrical Engineering, Qatar University, Doha 2713, Qatar; d Department of Electrical Engineering, Engineering Institute of Technology, Melbourne, Australia

**Keywords:** Index terms—plus-shaped slot, Patch antenna, DGS, 5G, Sub-6 GHz, WiMAX

## Abstract

A slotted plus-shaped patch antenna (PSPA) with defected ground structure (DGS) is modelled and proposed for 5G **Sub-6** GHz and WiMAX applications by using computer simulation technology (CST) MWS suite. The PSPA incorporates a rectangular slotted plus-shaped metal patch and a DGS. The **PSPA is designed on a Rogers RT5880 (lossy) substrate with a compact dimension of 20 × 35 × 0.79 mm**^**3**^. Its reflection coefficient is -52.06 dB resonating at 3.12 GHz and operates over a wider bandwidth of 2.56 GHz (2.67–5.23 GHz) to accommodate suitable **Sub-6 GHz bands. The PSPA has a good gain (2.44 dB), directivity (2.53 dBi)**, and VSWR (1.005) at 3.12 GHz with omnidirectional radiation characteristics. The maximum efficiency of the proposed PSPA is about 98% for almost loss free power radiation. The apex of estimated gain and directivity are 4.65 dB and 4.95 dBi. The impact of different physical parameters **on the antenna performance has** also been studied and analysed in this paper. Initially, the proposed PSPA has been investigated by using time domain (TD) solver of CST then again it is buttressed by applying frequency domain (FD) solver of CST. Furthermore, the design has also been verified by high-frequency structure simulator (HFSS) as well as FEKO (a computational electromagnetics software). All the simulators show a very good agreement in results.

## Highlights

• A slotted plus-shaped compact patch antenna is modelled and proposed for 5G Sub-6 GHz/WiMAX Applications.
• The designed antenna possesses a very low reflection loss (-52.06 dB) and wide bandwidth of 2.56 GHz ranging from 2.67 GHz to 5.23 GHz.
• The antenna is highly efficient (maximum efficiency is about 98%) with a peak gain of 4.65 dB.
• The proposed design is validated by both time domain and frequency domain analysis.

## 1 Introduction

The world is facing the technological revolution in the 21st century which introduced a redefined civilization of global connectivity. Wireless communication is one of the key features of this technological revolution, which has a great impact on our daily life. To fulfil the key performance factors of wireless communication such as higher data traffic, huge connectivity, low latency of various wireless devices, the Sub-6 GHz technology is a suitable candidate. The Sub-6 GHz wireless bands are assigned for multiple wireless technologies like WiMAX, WiFi, 3G, 4G and most promising 5G applications [1, 2]. Sub-6 GHz has opened the door of a new era of innovation and connectivity which enables cloud computing, smart traffic system, AI services, automated industrial infrastructure, robotics, HD live streaming, virtual reality, augmented reality, space and astronomy, smart-home, smart transportation [3], IoT, remote education and health services specially during a period of global pandemic like Covid-19 [4]. Recently, some researchers are using machine learning approaches to design low cost portable electronic devices for 5G, WiFi, WiMAX, WLAN etc. applications [5]. To provide these numerous services, Sub-6 GHz 5G has some key features such as huge data rate, numerous connectivity, ultra-low latency, higher reliability, wider coverage area and higher mobility [6, 7]. Similarly, due to having the properties of a very high peak data rate, higher mobility and multi-device connectivity, WiMAX technology is used widely. The most popular band of WiMAX is 3.5 GHz (3.3–3.6 GHz) which lies in the Sub-6 GHz bands [8]. WiMAX technology is widely used for cellular backhaul, broadband internet, VoIP, interactive gaming, IP multimedia subsystem (IMS) etc. Though mm-wave 5G provides very fast data rate with high spectrum, the Sub-6 GHz 5G technology is more practical ready-to-go technology with its wider coverage and implementation capability. The most popular Sub-6 GHz 5G band is the 3.5 GHz band of (3.3–4.2 GHz). Various countries have deployed or licensed Sub-6 GHz 5G with different spectrum shown in Table 1 [9, 10].

**Table 1:** Sub-6 GHz 5G frequency bands for various areas.

Area	Range (GHz)
USA	3.7–4.2
Europe	3.4–3.8
China	3.3–3.6 and 4.8–5
Japan	3.6–4.1 and 4.5–4.9
Korea	3.4–3.7 and 3.7–4
India	3.3–3.6
Australia	3.4–3.7

In wireless technology, antennas are the key element to set up a communication link. Numerous researchers are developing various types of antennas all over the world. Microstrip patch antennas are the most popular type of antennas for their important features. Microstrip patch antennas have some desirable features like simplicity, robustness, compatibility of integration, cost effective, energy efficient, light weight and ease of fabrication. These salient features of microstrip patch antennas (MPAs), make them a hot cake of Sub-6 GHz wireless communication. They are evolving with independent shapes of numerous designs by the modern-day researchers with respect to their usability and requirement. Different methods are used to design a MPA, among which DGS is one of the popular strategies to enhance the radiation characteristics of the MPA [11]. DGS is a very popular technique for its versatility and independence of structure [12]. Through the years, different types of antennas have been developed to follow the increasing demand in numerous applications of wireless technologies. Some notable works for Sub-6 GHz and WiMAX applications are introduced in [13, 14, 15, 16, 17, 18, 19, 20, 21, 22, 23, 24, 25, 26, 27]. In [13], a circular shaped planner antenna is discussed for Sub-6 GHz wireless applications. The planar antenna covers a wider bandwidth of 3.05–5.82 GHz with its simplest structure without any lumped element. The rectangular slot on the ground improves the impedance matching. Despite its simplicity and omnidirectional radiation, it shows comparatively lower average gain and efficiency. A compact F-shaped slotted MPA is modelled in [14] for WLAN/WiMAX. The F-shaped antenna is modelled on FR4 substrate with a concise dimension of 19 × 25 mm^2^. The rectangular patch with two F-shaped slots and a circular patch at the ground plane facilitate the triple band operation with a proper impedance matching. For WLAN, WiMAX and Bluetooth applications, a fork-shaped antenna is proposed and fabricated in [15] with a dimension of 24 × 35 mm^2^. It is incorporated with a T-shaped ground plane, an I-shaped monopole and two branch lines which act as a cavity resonator for dual band operation where the gain and directivity are unspecified. In [16], a stepped patch antenna is introduced with multiple slots which improves capacitive effect and facilitates wider coverage of 2.4 GHz. Though it is designed and fabricated with a compact dimension of 20 × 30 mm^2^, it shows comparatively lower average gain (2.35 dBi) and low efficiency (74.7%). A line stripe ultra-wideband antenna with dielectric resonators is demonstrated in [17] for 5G applications. The rectangular resonators enhance the efficiency of the antenna despite its miniaturized structure. The sawtooth orientation at ground plane ensures proper impedance matching for higher order radiation modes. In [18], a compact double band antenna with rectangular slot and inverted U-structured stub is described for 5G Sub-6 GHz applications. It provides a high maximum gain of 7.17 dBi in spite of its smaller dimension (36 × 31 mm^2^) but the bandwidth is very narrow. It shows optimum specific absorption rate (SAR) value but using dielectric back cover has a huge impact on its performance parameters. A ring-shaped slot antenna with circular polarization is proposed in [19]. It is designed on FR4 substrate with an L-pattern feeder and circular slotted ground plane. The coupling between the feeder and circular slotted ground plane is done by radial and narrow slots. The circular polarization is ensured by slits on the ground plane for very narrow bandwidth operation. A planner antenna with circular-shaped patch and long fed-line is introduced in [20] for 5G wireless applications. Though it has the simplest structure with a partial ground plane, it covers a wider bandwidth of 2.9 GHz. It shows moderate gain and at the ground plane two rectangular parasitic elements are used to enhance the impedance matching as well as frequency shifting. In [21], a Circular ring microstrip patch antenna is proposed with a dimension of 30 × 30 mm^2^. To boost up the gain and return loss property of a multiband MPA, four circular rings are used with a partial ground plane. The multiband MPA has an apex gain of 4 dB for WiMAX applications. For 3.5 GHz 5G applications, 3 distinct elliptical patch antennas are studied in [22] where all of them resonate at 3.5 GHz with different radiation performances. The overall dimension and slots at ground plane differentiate their performance parameters.

In this paper, a PSPA is demonstrated for Sub-6 GHz and WiMAX applications. The proposed PSPA is mounted on Rogers RT5880 substrate with a concise dimension of 20 × 35 × 0.79 mm^3^. It covers a wider bandwidth of 2.56 GHz (2.67–5.23 GHz) with a high peak gain, directivity, good current distribution and efficiency for Sub-6 GHz and WiMAX operation. The article is further structured as follows: structure of PSPA is discussed in section 2, simulation, discussion and design validation are incorporated in section 3. At last, significant findings and contributions of the work are expressed in section 4.

## 2 Structure of slotted plus-shaped antenna

The slotted PSPA with a DGS is optimized and estimated radiation performance parameters using CST-MWS suite 2018. The proposed PSPA is designed on Rogers RT5880 (lossy) material (ε_r_ = 2.2, tangent loss = 0.0009 and thickness = 0.79). To design the rectangular slotted plus-shaped patch, feed line and ground plane along with a DGS, copper (annealed, thickness = 0.035 mm) material is used. Initially, the size of the antenna has been estimated by using some fundamental Eqs. (1), (2), (3), and (4) then the greatness of the MPA is optimized to 20 × 35 × 0.79 mm^3^ by using CST-MWS.

(1) $$Patch\;width,W_{P\;}=\frac{c}{2f_{r}}\sqrt{\frac{2}{\varepsilon_{r}+1}}$$

where, *c* = Velocity of light, *f*_*r*_ = Resonance frequency and

*ε*_*r*_ = Dielectric constant

(2) $$\text{Length},L=0.412h\frac{\left(\varepsilon_{reff}+0.3\right)\left(\frac{W_{p}}{h}+0.264\right)}{\left(\varepsilon_{reff}-0.258\right)\left(\frac{W_{p}}{h}+0.8\right)}$$

(3) $$\text{Effective}\;\text{length},L_{eff}=\frac{c}{2f_{r}\sqrt{\varepsilon_{reff}}}$$

and

(4) $$\text{Patch}\;\text{length},L_{p}=L_{eff}-2\Delta L$$

**Figure 1:**
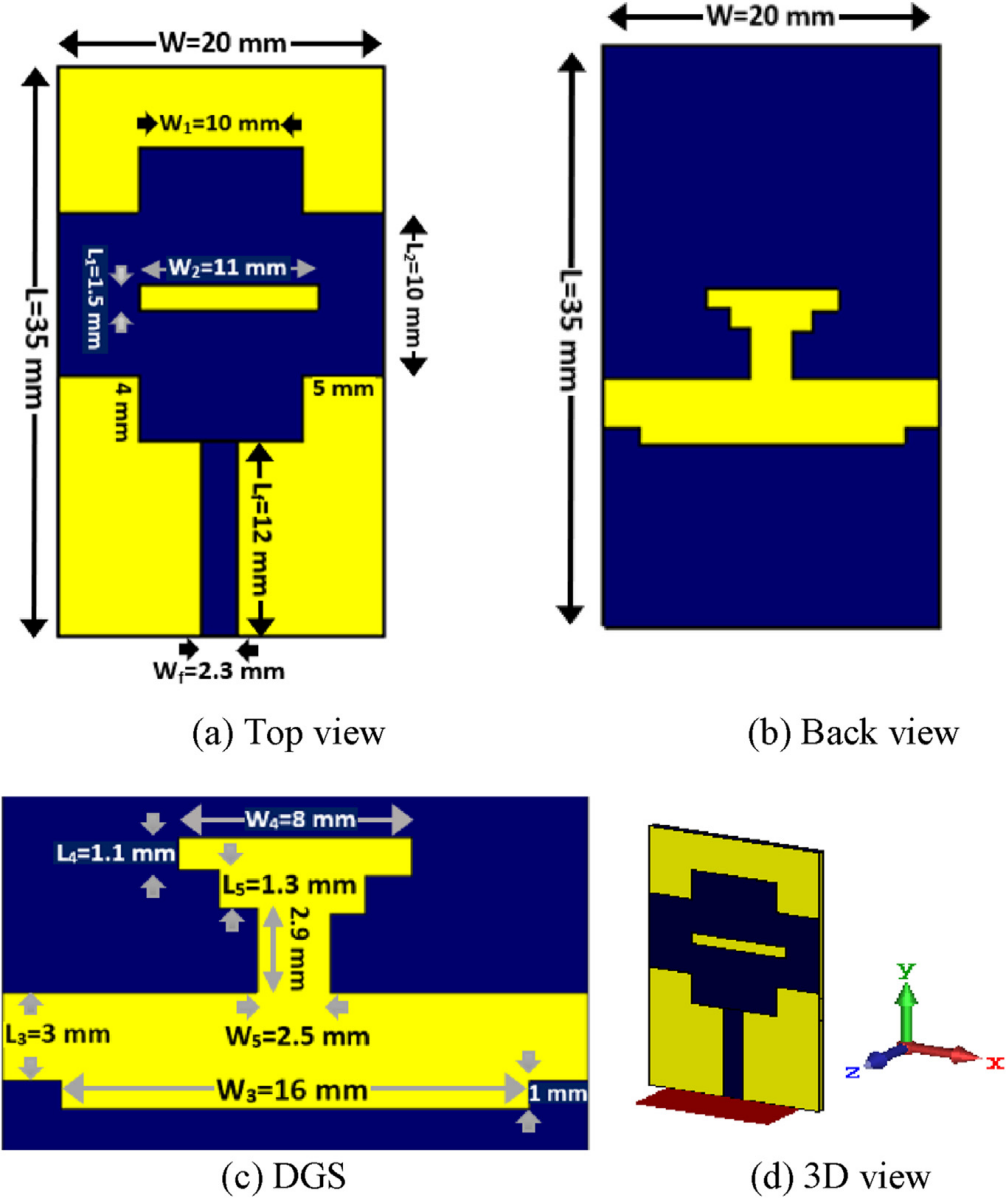
A slotted PSPA with a DGS.

The schematic front view and back view of the structure of the designed PSPA with a defected ground structure (DGS) is presented in Figures 1(a) and 1(b). The PSPA is fed by a feeder with a size of 12 × 2.3 mm^2^. The width of the four outer arms of the plus-shaped patch is 10 mm. The distance of the vertical arm from the edge of the horizontal outer arm is about 5 mm whereas the distance of the horizontal arm from the edge of the vertical outer arm is about 4 mm. The size of the slot of the radiating patch is 11 × 1.5 mm^2^. At the ground plane a DGS is introduced to improve impedance matching characteristics as well as for getting the required radiation performance. The labelling of the slot on the ground plane is shown at Figure 1(c) where a stepped T-shaped slot is designed with an arm of width 8 mm which improves the current distribution of the PSPA. At the lower portion of the DGS a 16 × 1 mm^2^ sized slot is etched. The schematic 3D view of the proposed PSPA for Sub-6 GHz/WiMAX application is shown in Figure 1(d). Initially, a rectangular-shaped planar antenna with full ground plane has been designed, then modified the patch to the proposed novel shape with a slot on patch and also incorporated a novel shaped DGS on the ground plane to get the frequency coverage of the intended applications with suitable radiation performance of the antenna. The DGS at the ground plane improves current distribution and proper impedance matching which makes the PSPA as a perfect contestant for Sub-6 GHz/WiMAX applications. All the design considerations of the PSPA are expressed in Table 2.

**Table 2:** Parameters’ list of the Designed PSPA.

Description and symbol	Value (mm)
Length (L)	35
Width (W)	20
Thickness (h)	0.79
Copper thickness (t)	0.035
Feeder length ($L_{f}$)	12
Feeder width ($W_{f}$)	2.3
Horizontal edge length of patch (L_2_)	10
Vertical edge width of patch (W_1_)	10
Width of slot on patch (W_2_)	11
Length of slot on patch (L_1_)	1.5
Width of lower slot on ground plane (W_3_)	16
Length of upper slot on ground plane (L_3_)	3
Width of upper arm of stepped T slot (W_4_)	8
Width of lower arm of stepped T slot (W_5_)	2.5

## 3 Results, parametric study and discussion

**Figure 2:**
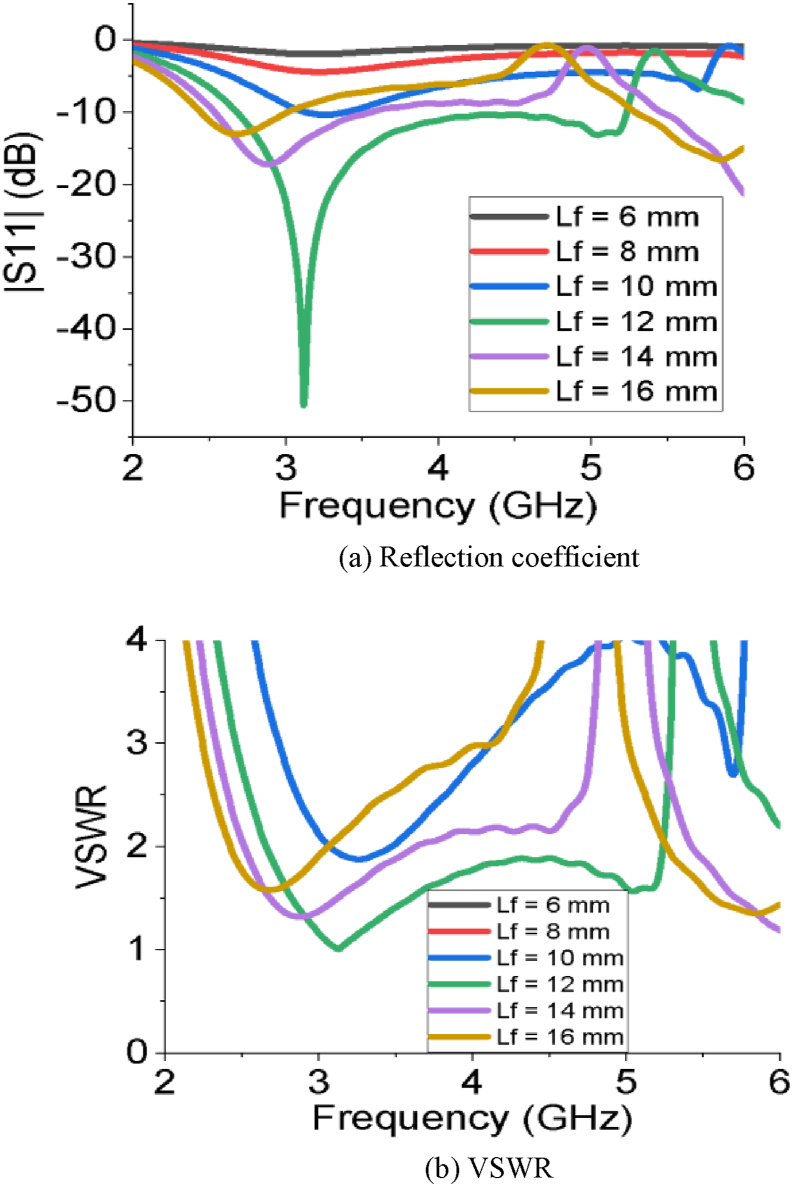
Impact of different length of feeder (L_f_).

**Figure 3:**
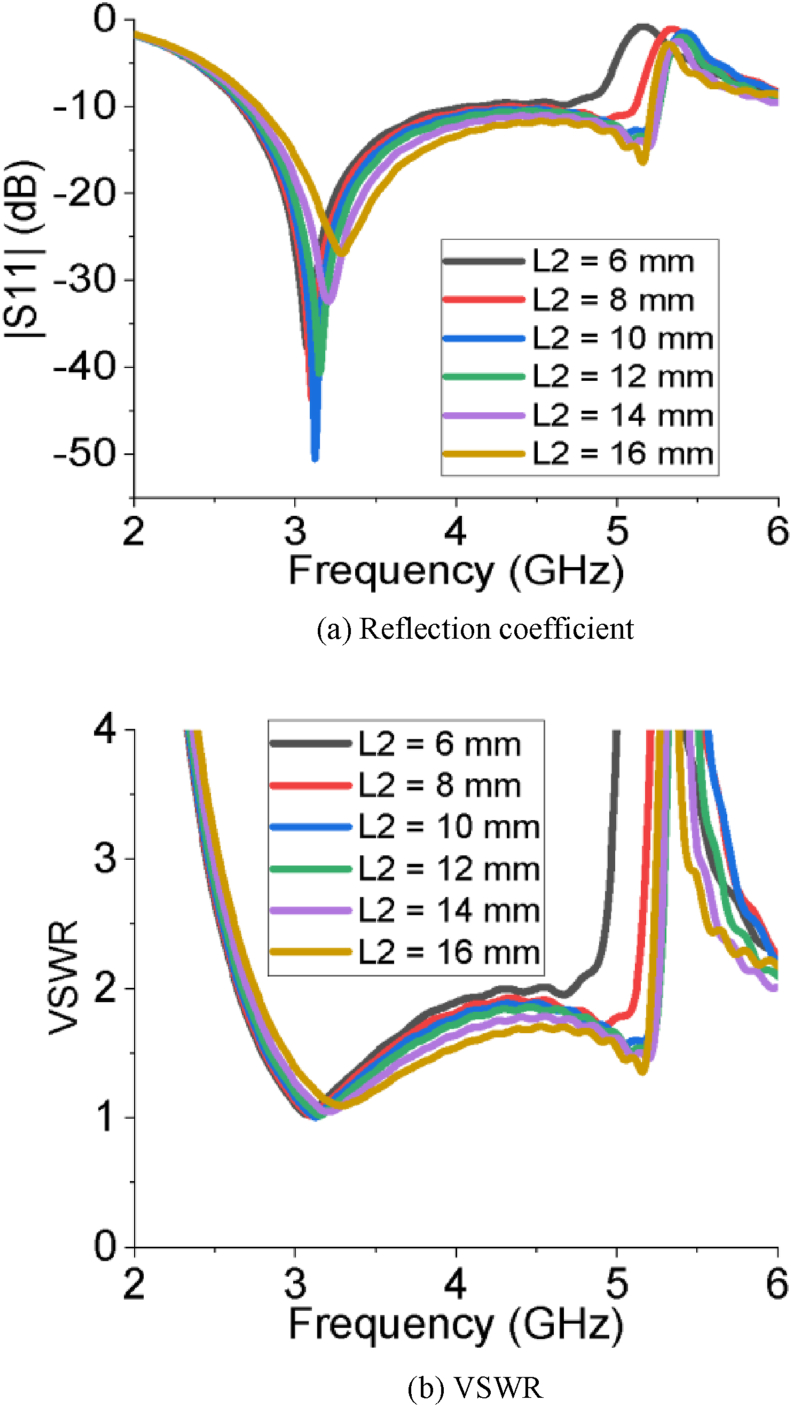
Impact of different horizontal edge length of patch (L_2_).

**Figure 4:**
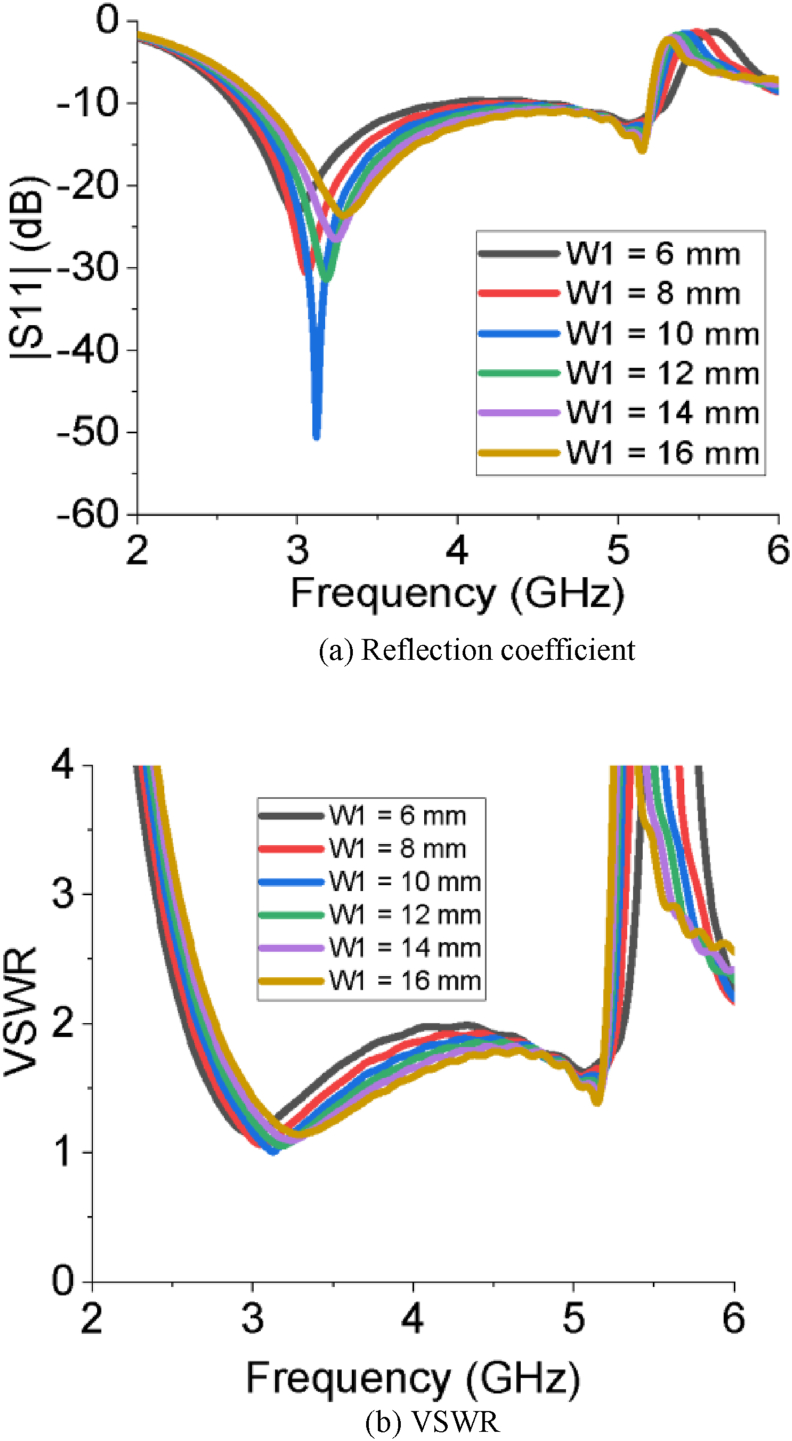
Impact of different vertical edge width of patch (W_1_).

**Figure 5:**
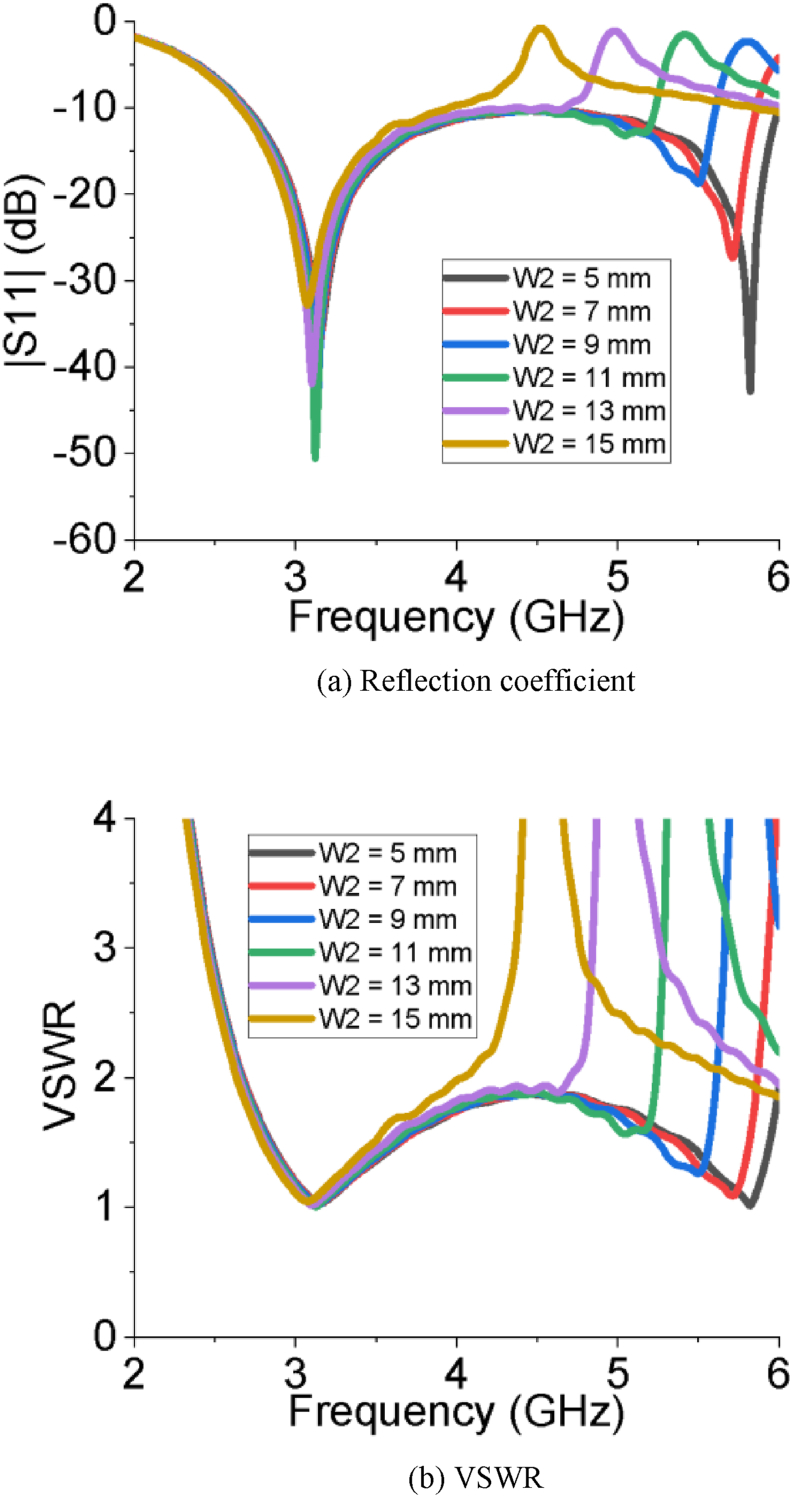
Impact of different width of slot at patch (W_2_).

The simulated results of all the performance parameters make the PSPA a perfect candidate for Sub-6 GHz and WiMAX applications. The PSPA covers a wider bandwidth of 2.56 GHz ranging from 2.67 GHz to 5.23 GHz with a superior reflection coefficient value of -52.06 dB. The slot at the radiating patch improves the reflection coefficient. The DGS structure at ground plane increases the bandwidth of operation. Figure 2 (a and b) represents the impact of the length of the feeder on the reflection coefficient and VSWR of the PSPA, respectively. The reflection coefficient and VSWR improve with increasing the length of feeder (L_f_) from 6 mm to 12 mm. After that further increment of the length of feeder (L_f_) influences to degrade the reflection coefficient as well as the VSWR. The impact of different horizontal edge lengths of patch (L_2_) on reflection coefficient and VSWR is also depicted in Figure 3. The length of L_2_ = 10 mm makes the designed antenna great in terms of reflection coefficient and VSWR. As per Figures 4 and 5, the lowest return loss and better VSWR have been achieved for vertical edge width of patch (W_1_) = 10 mm and width of slot at patch (W_2_) = 11 mm.

**Figure 6:**
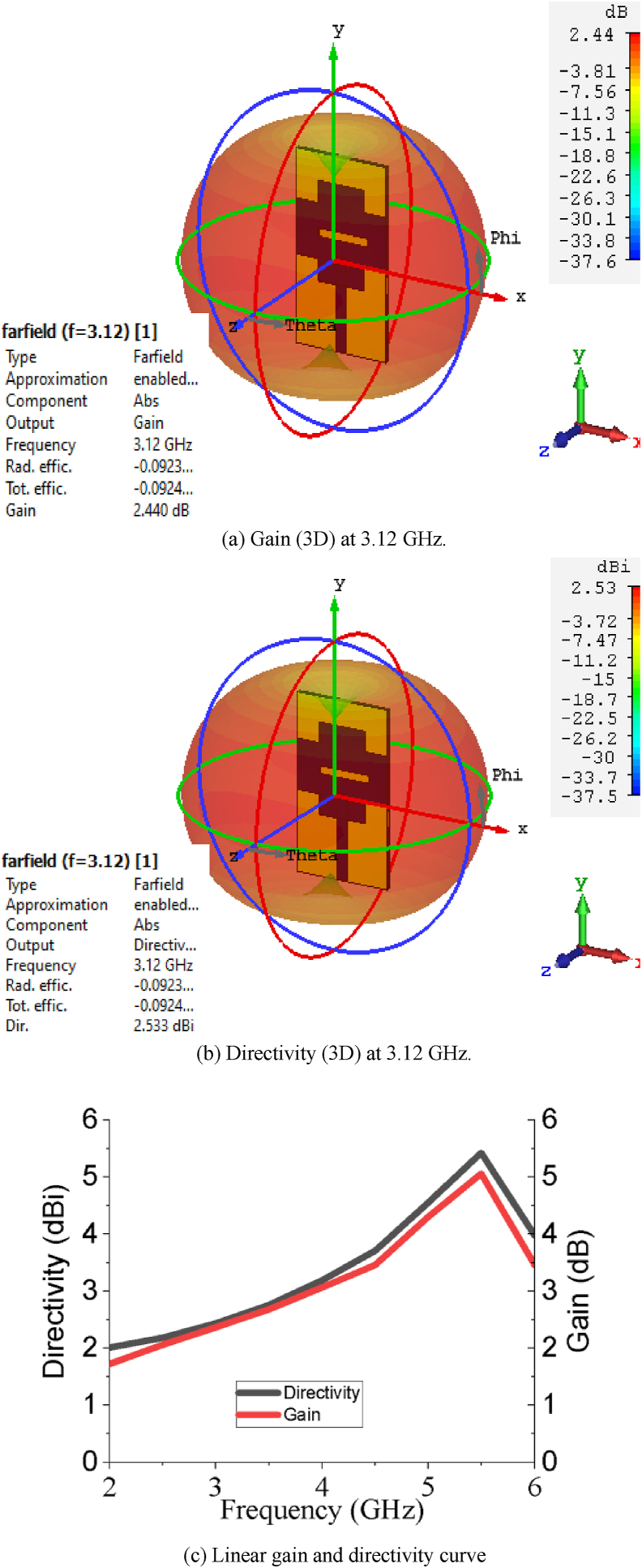
Gain and directivity of the designed PSPA.

The 3D gain and directivity at 3.12 GHz as well as linear gain and directivity graph are presented in Figure 6 to demonstrate gain and directivity performance of the proposed PSPA. The gain of PSPA at 3.12 GHz is 2.44 dB. Over the whole working band of PSPA, the gain varies from 2.17 dB to 4.65 dB. The antenna provides a higher peak gain of 4.65 dB for its optimized ground structure. The directivity of the PSPA is about 2.53 dBi at centre frequency 3.12 GHz. The directivity of the antenna over the whole operating band varies from 2.27 dBi to a higher peak directivity of 4.95 dBi. The length of the slot on patch (L_1_) and width of upper arm of stepped T slot (W_4_) do not have a major impact on reflection coefficient and VSWR. The value of VSWR at the centre frequency 3.12 GHz is 1.005 which is about unity and indicates minimal mismatch between antenna and the connecting feeder. Over the entire operating bandwidth, the VSWR value assures condition 1 < VSWR<2, which hints at a well optimized impedance matching of the PSPA.

**Figure 7:**
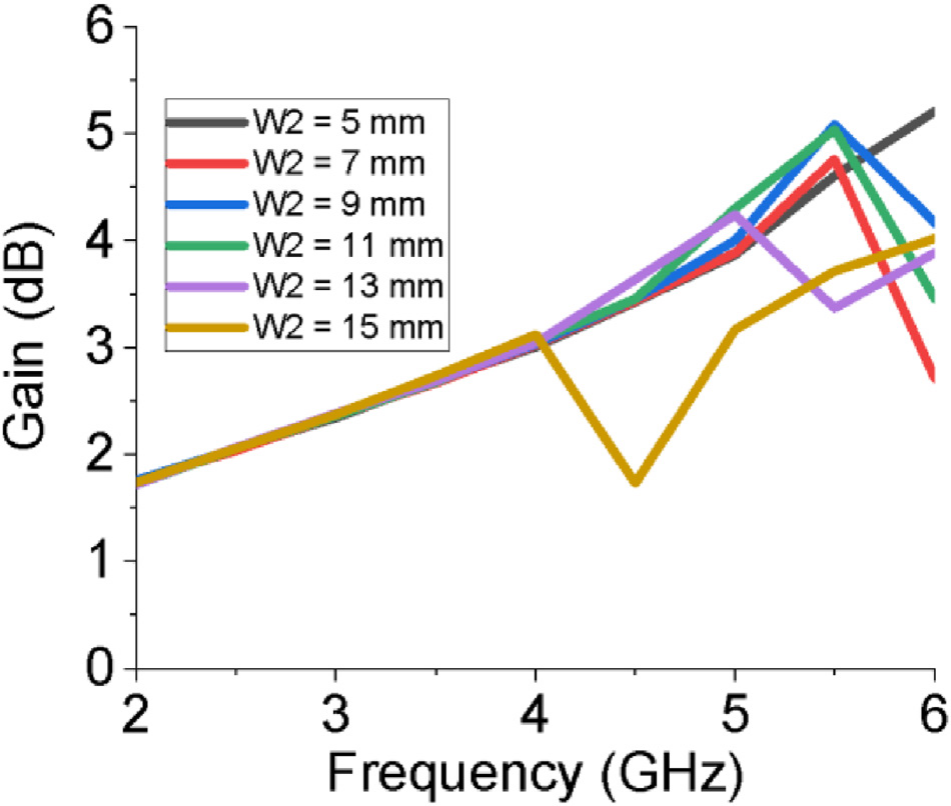
Gain for different width of slot at patch (W_2_).

**Figure 8:**
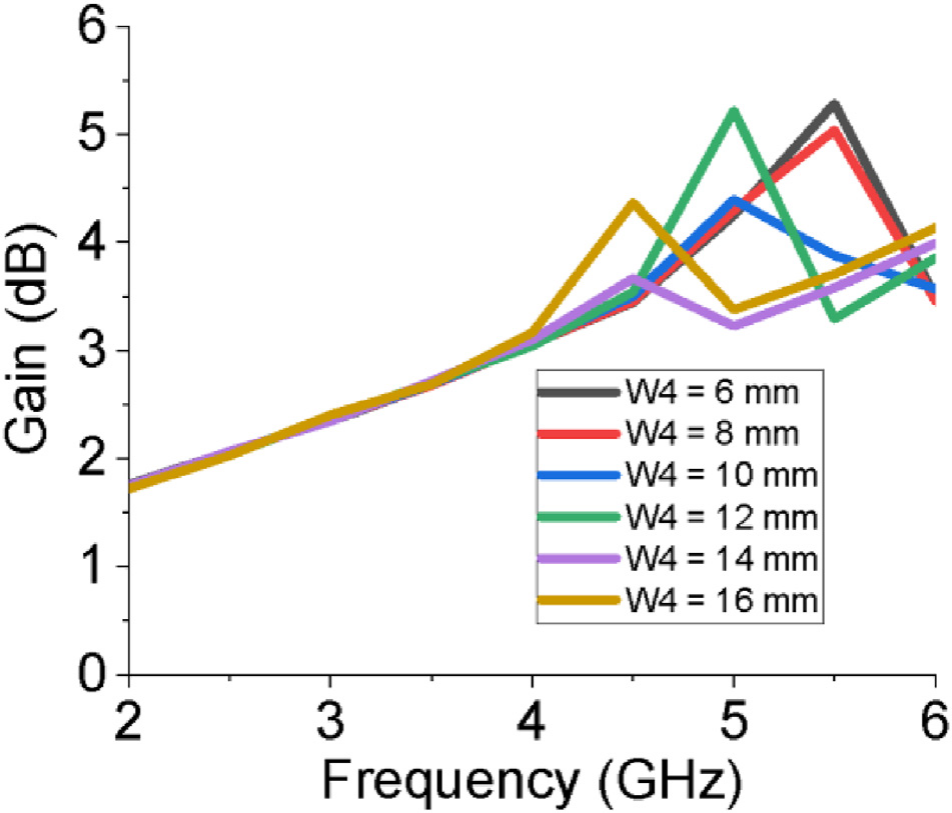
Gain for different width of upper arm of stepped T slot (W_4_).

**Figure 9:**
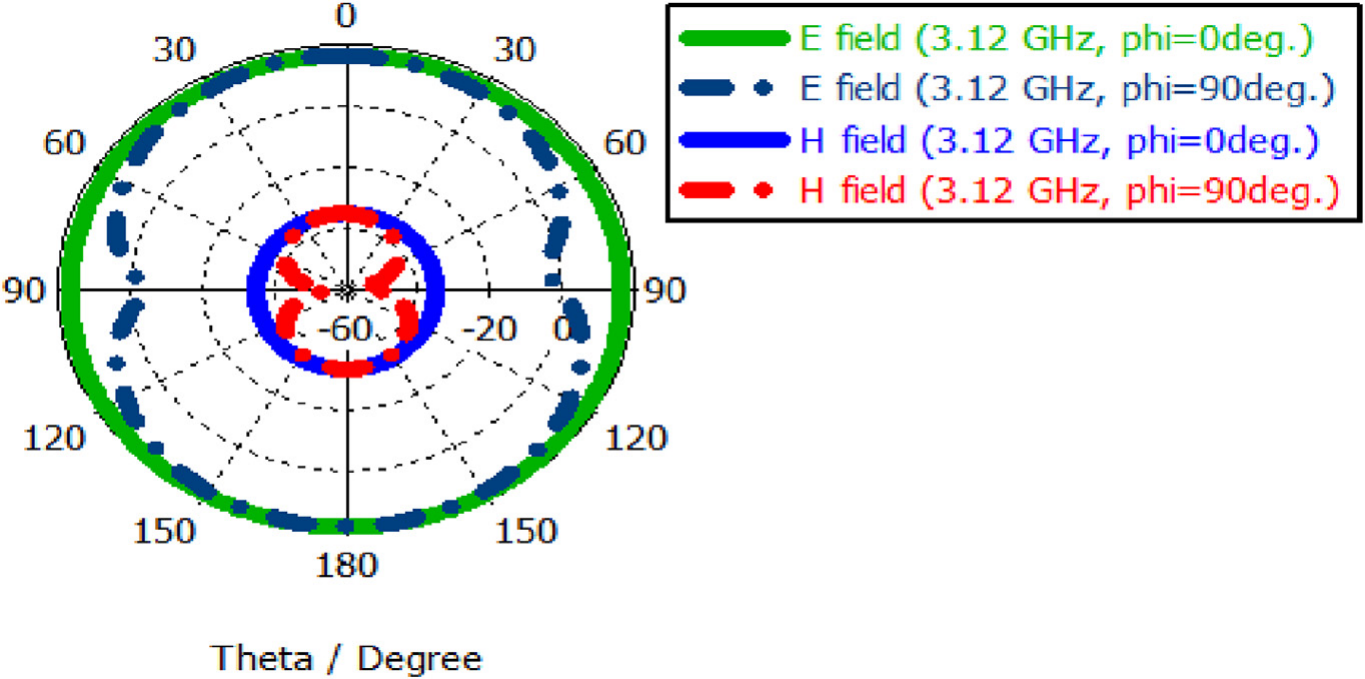
Fields (E and H) of the PSPA.

Though the length of feeder (L_f_), length of slot on patch (L_1_), horizontal edge length of patch (L_2_) and vertical edge width of patch (W_1_) do not have significant influence on the gain as well as directivity of the designed antenna, the width of slot at patch (W_2_) and width of upper arm of stepped T slot (W_4_) have notable impact on higher portion of the working band specifically above 4 GHz as shown in Figures 7 and 8. The radiation pattern of both field (E and H) of the PSPA in terms of polar plot are presented in Figure 9. The field patterns are expressed for azimuth angle φ = 0° and 90° at the middle working frequency of 3.12 GHz. The major lobe directions at φ = 0° and 90° are about 180° and 179° for both electric and magnetic field pattern. In terms of electric field pattern (E-Field), the major lobe magnitude for both azimuth angle φ = 0° and 90° are 17.3 dBV/m and 17.4 dBV/m respectively. In terms of magnetic field pattern (H-Field) the main lobe magnitudes for azimuth angle φ = 0° and 90° are both -34.2 dBA/m. The 3 dB angular width or HPBW at φ = 90° for both Fields (E and H) is 85.9°. The radiation properties of the slotted PSPA makes it an intended contender of required Sub-6 GHz applications.

**Figure 10:**
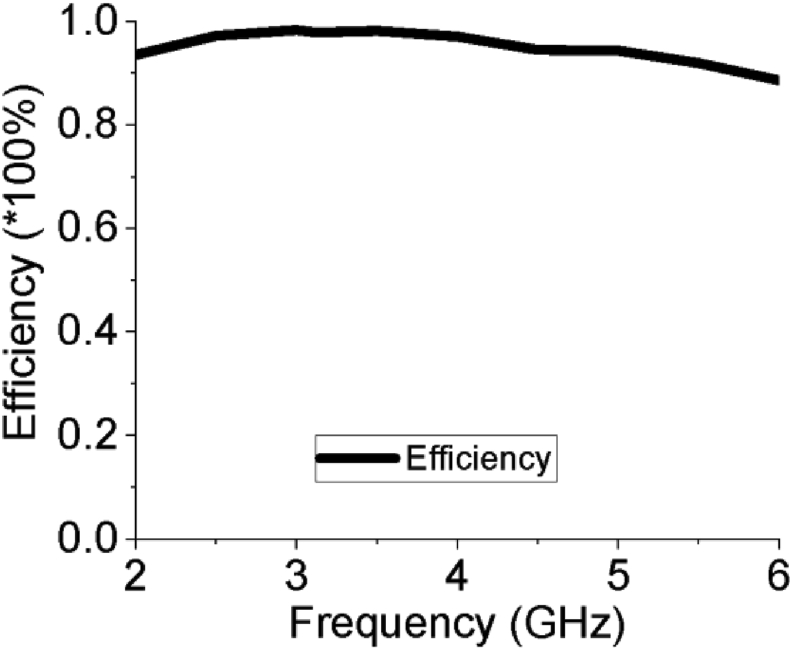
Radiation efficiency of the PSPA.

**Figure 11:**
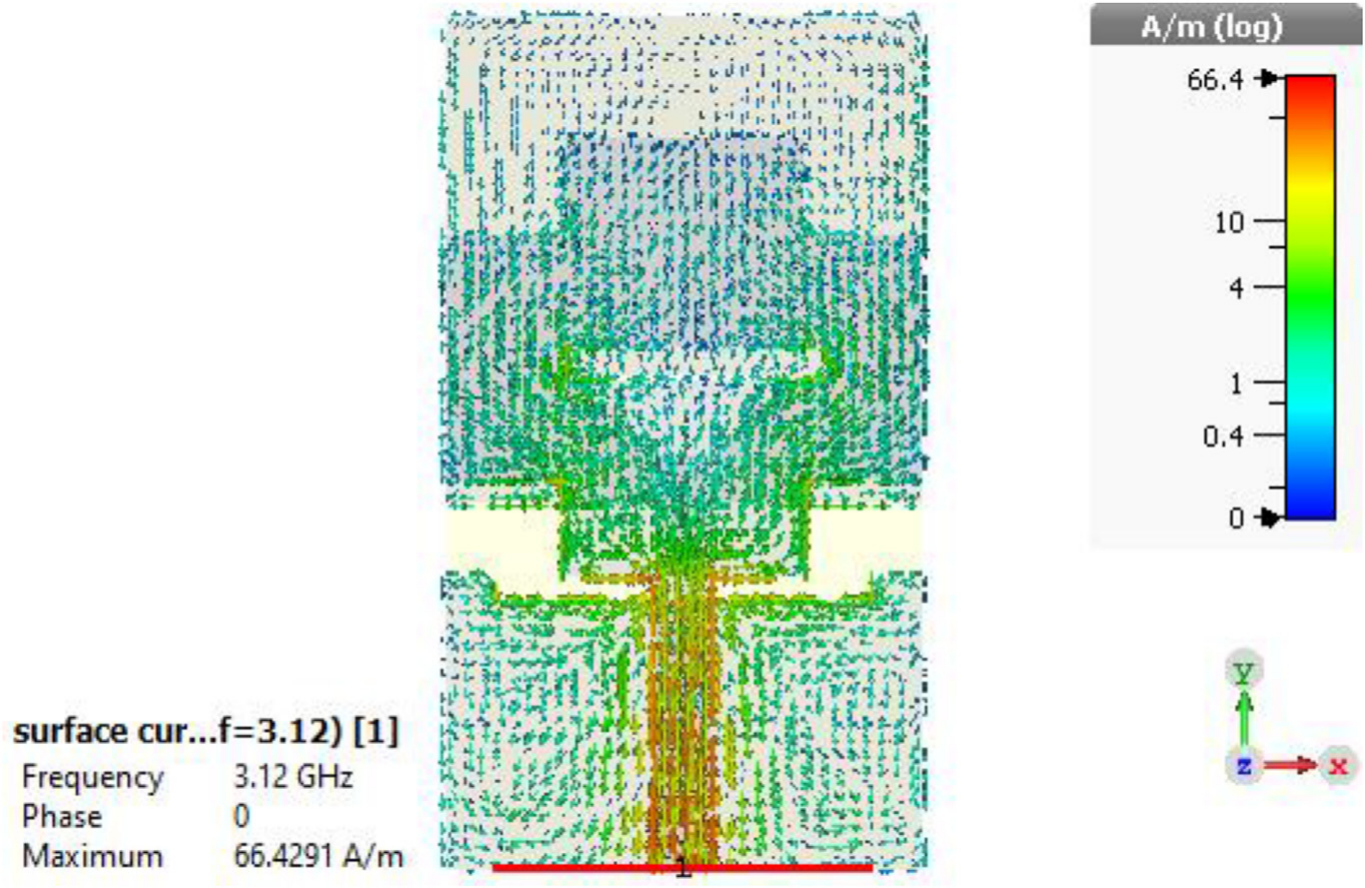
Current distribution of the PSPA at 3.12 GHz.

**Figure 12:**
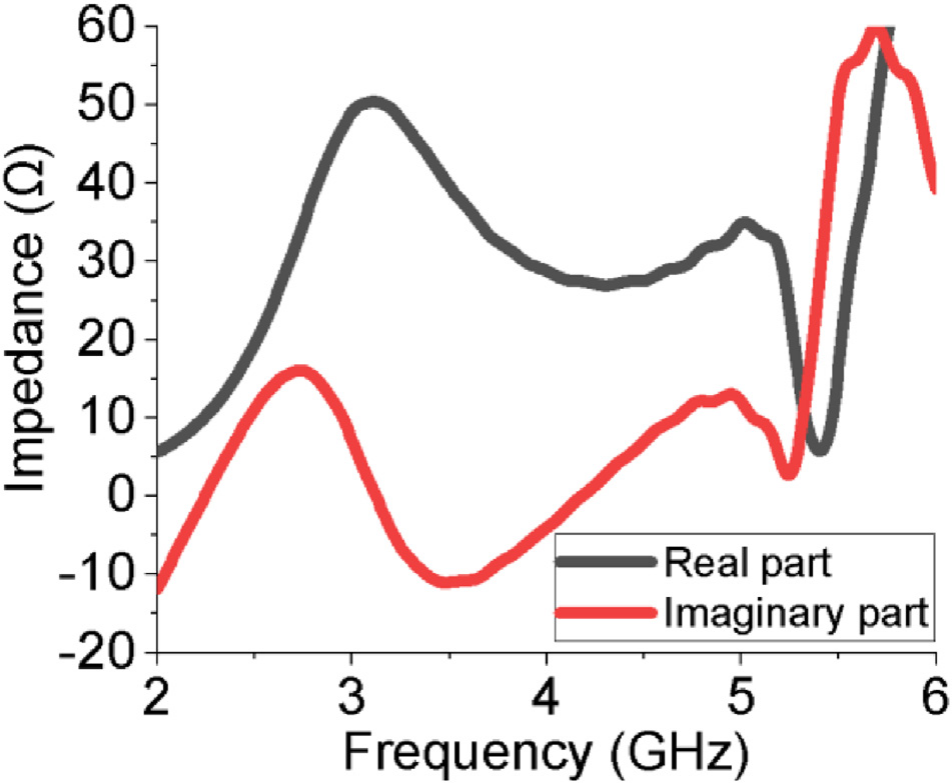
Z – parameters.

**Figure 13:**
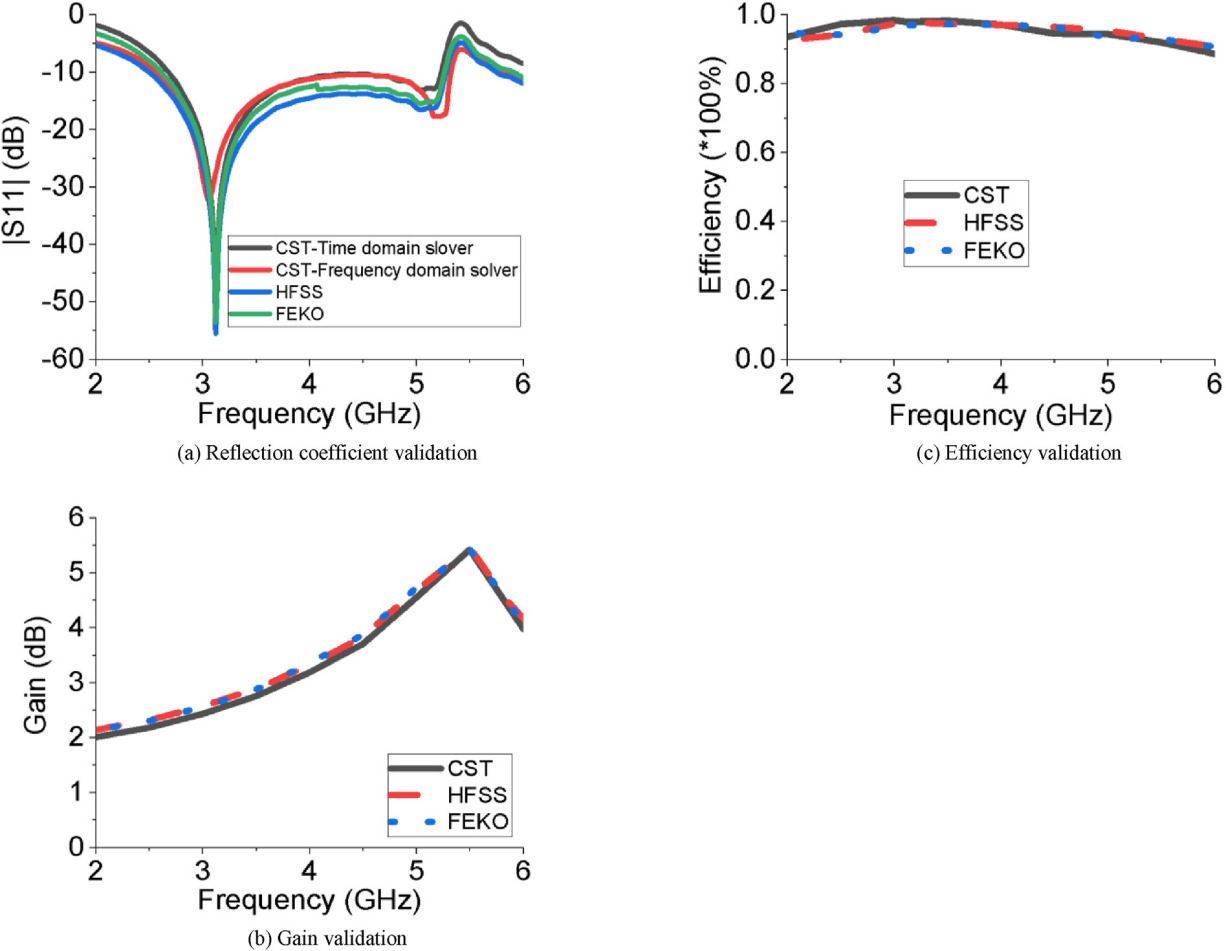
Design validation by using CST, HFSS and FEKO.

The efficiency of a MPA ensures how efficiently the antenna radiates the power fed to it and it is one of the key performance factors for any antenna. The efficiency curve of the slotted PSPA is paraded in Figure 10, where the average efficiency is approximately 95% which varies from 93% to a higher peak value of 98% over the entire bandwidth. This very high radiation efficiency of 98% increases its power radiation capability for required wireless applications as it radiates approximately all the power fed to it. The surface current distribution of the proposed slotted PSPA is displayed in Figure 11. The proper distribution of surface current over the whole PSPA is aided by the DGS at ground plane along with slots at the radiating patch. The maximum value of surface current is about 66.43 A/m. The current density is comparatively higher at the feeder of the slotted antenna. In Figure 12, the Z-parameters curve of the designed slotted PSPA for both real and imaginary parts are presented. At the centre frequency, the real part value is 50.33 Ω which is very close to standard 50 Ω. On the other hand, the imaginary part value at centre frequency 3.12 GHz is 0.14 Ω which is near the standard 0 Ω value. These values of Z-parameters ensure the proper impedance matching of the proposed slotted PSPA with a standard impedance of the port. The performance of the slotted PSPA has also been validated by applying both time domain and frequency domain solver of computer simulation technology (CST), high-frequency structure simulator (HFSS) as well as FEKO (a computational electromagnetics software) as presented in Figure 13. In CST, time domain (TD) solver uses finite difference time domain (FDTD) method and frequency domain solver uses finite element method (FEM) to solve Maxwell's equation. These two solvers use two different methods to solve the same problem. Both TD and FD solvers are originally from the finite integration technique (FIT) which acts on the integral formulation of Maxwell's equations. Therefore, if the antenna design is correct and all the parameters are well defined, it is pertinent that both TD and FD solvers provide similar results. Antenna characteristics related to port, reflection coefficient (S_11_ curve) validation is presented in Figure 13(a). There are only magnitudes of return loss slightly different but bandwidth remains almost same in case of all simulators. Antenna radiation characteristics, gain and efficiency, validation have also been incorporated in Figure 13(b) and Figure 13(c) respectively. There are very good agreements among all of them. Therefore, all the different analytical approaches of the proposed design show a very good agreement which ensured the design accuracy index. A comparison of our work with some recent relevant works are shown in Table 3. Though our proposed PSPA has a somewhat higher volume than designs described in [13] and [16], the performance of our antenna is better than theirs. Our designed slotted antenna shows minimum return loss, maximum gain and bandwidth with respect to all others which are the strengths of the proposed design for the intended applications. We have used comparatively low loss dielectric material, Rogers RT5880, to construct the proposed PSPA. The Rogers RT5880 is comparatively expensive than FR4, therefore, the fabrication cost will be somewhat higher.

**Table 3:** Comparison table.

Parameter	Reference No.									This work
	[13]	[16]	[18]	[22]	[23]	[24]	[25]	[26]	[27]	
Size (L × W) mm^2^	20 × 28	20 × 30	36 × 31	46 × 26	34 × 34	78 × 58	48 × 35	40 × 30	36 × 36	20 × 35
Electrical size	0.20λ × 0.28λ	0.21λ × 0.32λ	0.39λ × 0.34λ	0.48λ × 0.27λ	0.35λ × 0.35λ	0.48λ **×** 0.64λ	0.3λ × 0.22λ	0.44λ × 0.33λ	0.34λ × 0.34λ	0.18 λ× 0.31 λ
Substrate material	FR4	FR4	FR4	FR4	FR4	PET	FR4	FR4	FR4	Rogers RT 5880
Centre frequency (GHz)	4.8	4.71	3.3, 4.5	3.5	3.4	4.28	2.1, 3.5	3.41, 3.83	3.47	3.12
Reflection coefficient (dB)	≈ -28	-32.6	≈ -22, -23	-20.44	≈ -22	≈ -25	≈ -38, ≈ -22	-26.17, -31.15	-23	-52.06
Maximum Gain (dB)	2.45	2.69	7.17	4.42	3.8	3	4.044	2.5	4.08	4.65
BW (GHz)	2.77	2.4	0.34, 0.81	2.15	0.65	2.01	1.95	0.72	1.01	2.56
Maximum Efficiency (%)	87.13	79.6	≈80	NA	NA	≈80	92	NA	NA	98
Applications	5G Sub-6 GHz	LTE, 5G Sub-6 GHz	5G Sub-6 GHz	5G	5G	5 G Sub-6 GHz, WLAN	4G, 5G, NB-IoT	5G Sub-6 GHz	5G	5G Sub-6 GHz, WiMAX

• NA = Not available.

## 4 Conclusion

A slotted plus-shaped antenna with a DGS is demonstrated in this article for 5G Sub-6 GHz and WiMAX applications. The rectangular slot on the patch and a DGS on the ground plane enhance its radiation performances for the intended communication systems. The proposed PSPA has an excellent reflection coefficient (−52.06 dB) at 3.12 GHz. It handles over a wider bandwidth of 2.56 GHz. The VSWR value of the slotted PSPA fulfils 1 < VSWR<2 over the entire bandwidth which ensures proper impedance matching of the proposed PSPA. In spite of its simplest structure and compact size (20 × 35 mm^2^), it shows higher gain, directivity and efficiency with omni-directional radiation characteristics for Sub-6 GHz wireless communication. Different parametric studies have also been investigated and analysed in the work. The performance of the PSPA has been validated by using CST (both TD and FD solvers), HFSS and FEKO. All the results obtained from all three different electromagnetic simulators show a very good agreement which ensures the accuracy of the proposed design. An extensive comparison with some relevant published works also indicates the strong points and utility of the proposed design for the indented applications. Therefore, the slotted PSPA can be a suitable candidate for 5G Sub-6 GHz and WiMAX applications.

## Declarations

### Author contribution statement

Liton Chandra Paul: Conceived and designed the experiments; Performed the experiments; Analyzed and interpreted the data; Wrote the paper.

Sajeeb Chandra Das: Performed the experiments; Analyzed and interpreted the data.

Tithi Rani: Conceived and designed the experiments; Performed the experiments; Wrote the paper.

S. M. Muyeen: Analyzed and interpreted the data; Contributed reagents, materials, analysis tools or data.

Sk. A. Shezan: Performed the experiments; Analyzed and interpreted the data; Contributed reagents, materials, analysis tools or data; Wrote the paper.

Md. Fatin Ishraque: Conceived and designed the experiments; Analyzed and interpreted the data; Wrote the paper.

### Funding statement

The publication of this article was funded by Qatar National Library.

### Data availability statement

The data that has been used is confidential.

### Declaration of interest’s statement

The authors declare no conflict of interest.

### Additional information

No additional information is available for this paper.
